# Book Reviews

**Published:** 1813-10

**Authors:** 


### CRITICAL ANALYSIS
### OF RECENT PUBLICATIONS
IN THE
### DIFFERENT BRANCHES OF PHYSIC, SURGERY, AND MEDICAL PHILOSOPHY.

*Tracts on Delirium Tremens, on Peritonitis, and on some other internal Inflammatory Affections; and on the Gout.* By Thomas Sutton, M.D. of the Royal College of Physicians, late Physician to the Forces, and consulting Physician to the Kent Dispensary. 8vo. pp. 272. London, 1813. Underwood.

In noticing the diseases treated of in the present work, we shall pursue the order adopted by the author. The first complaint which claims our attention is a variety of phrenitis, with which disease, indeed, it has hitherto been confounded, so much so that Dr. Sutton, we believe, is the only writer who has regarded it as a distinct malady. He names it Delirium Tremens from an essential symptom of the complaint. Now, as we find nothing in the history of this affection to induce us to separate it from phrenitis, would not the term *Phrenitis è temulentia* be more appropriate than the one suggested by the author? In all the cases he has met with, drunkenness, or indulgence in liquor, mainly produced the complaint; and if Phrenitis be proper as the genus, *è temulentia* will mark the species. Delirium and tremor occasionally constitute symptoms of typhus fever, and several other diseases in their latter stages, and on that account, in our opinion, render the term *delirium tremens* objectionable to designate a mere variety of phrenitis. It, perhaps, will be alleged that the disease in question cannot be a species of phrenitis, because the treatment to be pursued in the two affections is directly opposite; but this argument has no weight with us, when we consider that there is hardly a disease of importance, or a variety of a disease, which does not require very different treatment in its progress, or as it appears in a particular patient. Bronchitis acuta is not to be treated like bronchitis asthenica.

The merit of Dr. Sutton, however, neither consists in describing a new disease, nor in calling an old one by a new name: it rests upon separating a series of symptoms produced by a certain cause, from a well-known complaint, with which they have been confounded, and bad practice has in in consequence been generally pursued. It has been usual to treat delirium tremens (as the doctor would have us term the affection) as phrenitis; but, during his residence on the coast of Kent, he found two modes of treating the complaint prevailed,—the one by bleeding and depletion, the other by opium. The latter practice was the most successful, but the practitioners who had adopted it could assign no reason for the faith within them: they administered it because they found it useful. "I know, from experience, (said a physician to whom Dr. Sutton applied for information on the subject) that opium is of great use in this disease, and that when sleep is procured, the patient more frequently gets better; but I have nothing to guide me to form an opinion from, as to what may be the state of the brain, nor in regard to the *modus operandi* of the remedy, than that the measure of its beneficial efficacy is by procuring sleep."

The history of this complaint, as related by the author, is highly interesting: we have already noticed it in our Half-yearly Report of the Progress of Medicine, in the present volume, page 17, to which we now refer.

This accurate account is followed up by several cases which illustrate the author's treatment, and detail of symptoms. In the first, the patient had labored under acute rheumatism for ten days; and for three days previous to Dr. Sutton's visiting him had been very delirious, and without sleep.

"When I saw him (the doctor writes) he had a strait waiscoat on; of course might be considered to have been very ungovernable. He had been bled in the course of the day, and the blood was buffy; the bowels had been acted on freely; a stimulating composition had been applied to the head, and a blister between the shoulders: notwithstanding which, all the symptoms had become worse. The pulse was very quick:* there were continual workings of the tendons, with considerable tremors, and profuse sweats. After making inquiry respecting the habits of the patient, and collecting all the information I judged to be necessary, I proposed to administer forty drops of laudanum in a draught every two hours, until sleep was procured. Three of these draughts were given in succession, when the patient

"* Dr. Saunders observed, that he knew no disease in which the pulse became so rapid, and recovery ensued, as in numerous cases of this affection; which perfectly accords with my observation: but the recoveries alluded to have been effected by the treatment which is about to be pointed out, the efficacy of which, and its preference to all others, Dr. Saunders has been confirmed in, from a long experience, and attentive observation. I never saw a case of recovery, when the pulse was very rapid, except by the employment of opium."

fell fell asleep, and continued so for some hours. I saw him again at the interval of sixteen hours: he was then collected; and, after recommending forty drops of tincture of opium to be given morning and evening, for some few days, I took my leave; and was happy to find, on future inquiry, that the patient recovered rapidly from that period."

We have not space to insert as many of these cases as we wish. The fourth is particularly interesting, because, for the first two days of the attack, the treatment had been conducted on the antiphlogistic and depleting plan, to some extent; but with an increase of the disease. On the third day, four doses of extract of opium in two-grain pills were given every two hours, when the plan not appearing to succeed, the case was considered hopeless; but on Dr. Sutton visiting the patient three hours after taking the last pill, he found him "in a profound sleep, the respiration quiet, the pulse full and regular, with neither tremor, subsultus tendonum, or hiccough; the contrary of which had been the case a few hours before. The patient was the next day completely tranquil and rational, and recovered without interruption."

The sixth case appeared so desperate and hopeless, that we shall insert it as an useful example to practitioners to resist the importunity of friends, which so often would have us quietly look on, and abandon a patient to his fate, without having recourse to vigorous practice.

"The patient was a robust young man, and much given to drinking of spirits. Two days previous to my seeing him, he had been bled largely, and blistered, and the bowels had been freely opened. Afterwards, opium had been given with tolerable freedom; notwithstanding which, the delirium had continued unabated, with uninterrupted sleeplessness. On my visit, it was agreed to give two grains of opium every two hours, until rest was procured. When I again saw the patient, he had, in the course of twelve hours, taken eight grains of opium; but as the friends were possessed with the idea of the impossibility of his recovery, they had, for several hours, laid aside the administration of medicine. During the interval of my visits, blisters had been applied to the calves of the legs. I now found the patient, after having been much exhausted by exertion, exceedingly restless, in a profuse clammy sweat, with tremors, a very constant subsultus tendonum, a pulse scarcely to be perceived, the countenance fallen, and the eyes muddy; the urine had been discharged involuntarily; and the patient was constantly occupied in picking the bed-clothes. Under such a state of things, the chances of recovery appeared to be little; but, as we were assured of the nature and origin of the disease, and had seen much beneficial effects from opium under some very unpromising circumstances of delirium tremens, it was determined still to advise its use, and to encourage the friends to hope that something favorable might yet ensue, by following lowing the directions that would be given. It was then directed to give two grains of opium every hour, until rest was procured, which happened after the fourth dose, and the patient had a tranquil sleep of some hours. The next day I found him rational, his pulse free and not quick, the subsultus tendonum gone, the tremors much diminished, and he discovered some inclination for food. The patient took six grains of opium, in divided doses, for some days, and got, in a short time, into good health."

We shall now forbear quoting more cases, having, we conceive, already stated enough of the author's practice to enable our readers to judge for themselves. We need hardly warn them that it is of the last importance to ascertain the peculiar nature of the complaint, before they give a remedy which, in the doses recommended by Dr. Sutton, would probably prove fatal in idiopathic phrenitis; and we trust the doctor will pardon us if we hint that he has not altogether enabled us by his account of it to distinguish delirium tremens as a distinct disease. He seems chiefly to rely upon his knowledge of the cause, indulgence in drinking; but this, in many instances, is extremely difficult to discover. Even in cases where the patient is actually intoxicated, we have known the affection to be mistaken by an eminent practitioner for typhus fever; and this disease again we have seen affect a patient so remarkably, that he has been supposed by pupils at an hospital to be drunk. Phrenitis indeed attacks more suddenly, and with greater violence in the commencement, than is the case in delirium tremens; but we all know how vaguely the history of the accession of a complaint, especially when the patient is insensible or delirious, is communicated to the physician, who does not see him till several hours, or days, after the indisposition has commenced. Tremor then must be the leading diagnostic symptom, but this occasionally is observed in phrenitis, and often is present in other diseases. Another small distinction too, Dr. Sutton mentions as distinguishing this disease from mania, for the two affections have occurred in the same individual. "The mind in delirium tremens is occupied and worried about private affairs." Dr. Saunders also, whose acuteness of tact is universally acknowledged, has pointed out the motion of the hands as characteristic in delirium tremens. "He has often considered the motion of the hands in this state of disease, as if the patient might, with imperfect vision, be searching for things, and occasionally rapidly catching or avoiding them: such, for instance, as if in search for rats or mice, being things he wishes partly to lay hold of, and partly to avoid."

(To be continued.)

### *Edinburgh Medical and Surgical Journal.*

(Continued from p. 243.)

### IV. *Case of Diseased Uterus, with the Appearances on Dissection.* By T. Salter, Surgeon.

The symptoms were not very different from those which usually accompany diseased uterus: there was no discharge per vaginam, and the os tincæ was found free from disease. After an endurance of lancinating pains for six years and upwards, the patient expired.

Examination *post mortem.* "On the posterior surface of the uterus were found two tumors. The diameter of the largest, at its base, extended from a longitudinal line drawn through the middle of the uterus to its right margin. The other was situated a little to the left side, and nearer to the fundus uteri, and was about the bigness of a common marble. The whole uterus appeared red, and more vascular than natural, and vessels were observed ramifying on the surface of the tumors. On examining the structure of the largest tumor, its superficies, an eighth of an inch in thickness, resembled in color and texture the other parts of the uterus. The central parts were of a light color, and very hard; but no ligamentous bands were to be seen. The os uteri was in a healthy state, and the cavity free from disease."

This disease was suspected to be syphilis and cancer, but was a case of slow inflammation, which, possibly, would have been checked in its progress, perhaps cured, by a judicious employment of evacuants.

### V. *Complicated Case of Cancerous Stomach.* By William Cooke, Surgeon.

This disease, which ended in the death of the patient, appears to have been excited or increased by a particular position, taken with more celerity, perhaps, than a woman at 82 could bear. The symptoms were constant soreness of the abdomen, continual pain about the umbilicus, sickness, constipation at first for some months, and some time afterward diarrhœa with tenesmus irregularly.

" *Sectio cadaveris.* The greater curvature of the stomach, a mass of omentum resembling the pancreas in size and shape, and the transverse arch of the colon were united together, and formed an indurated substance. The coats of the former viscus were so extremely tender in the lesser curvature, as to tear by the weakest effort. Its cavity was considerably diminished. The cardiac half of the villous coat was slightly thickened and inflamed. In that portion of the greater curvature nearest the pylorus, the coats were greatly thickened, except where they were destroyed by a large ulcer. The circumference of this ulcer was so thickened and puckered up, that it resembled a purse.

 When

When this margin was put on the stretch, it measured from two to three inches in diameter; but when allowed to contract, it did not exceed one inch. In its contracted state, the depth of the ulcer was about an inch and a quarter. In some places, the ulceration shot beyond the principal boundary, and destroyed the villous coat; and whenever this occurred, it was also encircled by a ridge, corresponding in appearance with that round the larger ulcer. At the bottom were several dark-colored sloughs, and two openings by which it communicated with the cavity of the abdomen. One of these was nearly large enough to admit the little finger within it, and the other was capable of receiving a goose-quill. In two places the small intestines were thickened. I removed one of these morbid parts, which occupied about an inch of the ilium. The coats on one side were at least half an inch thick, hard, and had two openings through them, sufficiently large to allow a probe to pass with facility. At the caput coli was a mass of disease as large as the fist, which, on examination, I found to arise from enlarged glands, situated between the peritoneal and muscular coats, and varying in size from a pea to a large wallnut. They were firm in structure, and variegated in appearance. The villous coat of this part was inflamed. The ulcer in the stomach extended to within half an inch of the pylorus, but did not affect the orifice in the smallest degree."

VI. *Case of Brain-fever following Intoxication, with some Observations.* By John Armstrong, M.D.

The disease, of which this case is a well-defined instance, has lately been examined with so much attention to its natural phenomena, with so little of the influence of hypothesis, and subjected to a treatment so generally successful, that it may be considered as an instance of the advance of a rational medical science. The case is related with minuteness, and, probably, precision; and the judicious use of stimulants, particularly opium, was followed by the most unequivocal benefit. One circumstance Dr. Armstrong dwells on with proper decision. It is the danger attending the employment of coercion or confinement in the furious delirium that accompanies this peculiar state of the brain.

VII. *On unusual Cases of Anorexy.* By B. Granger, Surgeon.

Some instances of extraordinary abstinence are here related, to countenance the possibility of the abstinence of Ann Moore. The subsequent explanation of this woman's case will supersede all observations upon them.

VIII. *On the dangerous Effects of Infusion of Tobacco administered in a Glyster.*

The power of the Nicotiana Tabacum we have frequently witnessed, but, like all other powerful remedies, it requires caution in the use. We have here a remarkable instance of the the deleterious operation of a dose generally recommended, and usually employed, in enema.

An infusion of ʒij of tobacco in ℥viij of boiling water, was injected into the bowels of a young man suffering under colic and constipation. "No sooner was it administered, than he was seized with convulsions, became speechless, and died in an hour or two." Permission to examine the body could not be obtained, but there was no doubt in the mind of the practitioner who prescribed this enema, that it was the direct cause of death.

IX. *Case of Hæmatirrhæa, or Petechiæ without Fever, which terminated fatally in the Hospital of the 6th Dragoon Guards, at Piershill near Edinburgh.* By E. WALSH, M.D.

James Curry, private in the 6th D. G., 30 years of age, robust, dark complexion, of sober habits, and healthy, was attacked, on the 27th of December, 1812, with pain in the head, rigors, and sickness. In the evening these symptoms disappeared. After having slept well, early on the morning of the 28th he was alarmed by a spitting of blood. On being taken into the hospital, there was observed "an eruption of petechiæ, numerous on the shoulders, across the breast, and on the neck, where they were not elevated above the cuticle; a few, more prominent, appeared on the forehead and temples, close to the hair; and some, of a still larger size, were seen on the hairy scalp. There were scarcely any discernible on the inferior trunk and extremities. These maculæ were of various magnitude; the largest not exceeding that of a pea. Their color was a dark purplish brown, having a resemblance to, but more prominent than, those which sometimes occur in typhus fever. On inspecting his mouth, petechiæ were found few in number, but large and distinct on the tongue, palatum molle, and as far down the throat as could be seen. From these there was a continual oozing of florid blood, which, mixing with the saliva, occasioned a constant bloody spitting." The pulse was 75, soft and full, no thirst, skin cool but dry; neither head-ach, pain, or debility: but there was weight at the stomach, and inclination to vomit; and the bowels were constipated.

He took a saline draught, in which was dissolved a grain and a half of tartarized antimony. This vomited him, and he threw up four quarts of a chocolate-colored fluid, intermixed with coagula of blood.

The 29th. He had slept, and perspired copiously in the night. At day-break he vomited, and threw up considerable quantities of a chocolate-colored fluid, mixed with fluid and coagulated blood. Petechiæ increased, now forming clusters on the trunk, and spreading over the lower limbs, most nu-

 merously merously on the legs. Pulse 86, bowels still constipated. In the course of this day the bowels being acted on, the fæces presented a mixture of grumous blood, and were extremely fetid.

The 30th. "On inspecting his mouth, the tongue appeared covered with a thick brownish-red crust, yet round the edges, and at the tip, it was moist, and of a natural color. Firm tenacious clots of blood adhered like polypi to the velum pendulum palati. The maculæ in the scalp had become large, black, and felt almost like warts. His muscular strength had suffered a great diminution; his pulse became feeble, and more frequent; a peculiarly heavy offensive smell came from his body; and his urine was intensely red without sediment."

At noon, on this day, he had a copious fetid stool, in which was much fluid blood: his features now sank; his right eye became tumid and black as from a blow, and the pupil of the left was contracted to a point; but his vision was perfect.

At four P. M. he had a violent fit of vomiting, in which he threw up a larger proportion of fluid blood than before. His strength now rapidly gave way: his pulse sunk, and became thready and intermittent.

At eight P. M. he was comatose; and by nine was in profound coma, with stertorous respiration. His pulse rose, was full, and 60 in the minute.

The 31st. He continued totally insensible, evidently in an apoplectic state, and expired at four this morning.

*Appearances on opening the body.*—The dark red pustules penetrated through the scalp, and left an impression on the cranium as if the head had been wounded with small shot. The right temporal muscle was black, as if severely bruised. On elevating the calvarium, a large coagulum of blood (a table-spoonful) fell out: it had pressed on the brain under the left temporal bone. The contiguous vessels of the pia mater were turgid, and black; but the superior lobes of the cerebrum were not in a diseased state. The liver was not diseased, but the spleen was unusually small. Large, but not numerous, petechiæ were scattered along the duodenum; some, but still fewer, were seen on the small intestines; and none were found on the arch of the colon. On the pylorus and anterior cavity of the stomach, petechiæ were numerous; but the great left curvature and superior orifice were so thickly covered with them, as to have the appearance of being sphacelated.

Both the remote and proximate cause of this rapidly-fatal case are equally obscure. It occurred suddenly to a robust and and healthy man, in the prime of life, without the intervention of any accident to which it could be attributed. Its seat was in the superior portion of the alimentary canal, gradually descending from the mouth downwards: it affected the urinary bladder, and ultimately extended to the brain, occasioning apoplexy and death.

X. *A remarkable Case of Dislocation of the Atlas.* By E. LAZZARETTO, Surgeon.

By violence, *ab extra*, the atlas was displaced from the foramen magnum; the corrugator-supercilii and occipito-frontalis were wounded. The symptoms were laborious breathing, rattling in the throat, dilatation of the pupil, slow and interrupted pulse, and paralysis of the lower exfremities. The atlas was replaced, and the man gradually recovered.

XI. *Case of Torpor of the Primæ Viæ, terminating fatally.* By J. GREEN, Surgeon.

This is a remarkable instance of a complete insensibility in the stomach and bowels to the stimulus of many highly-irritating substances, occurring in an athletic man of 25 years of age. After many efforts had been made to excite vomiting, to procure evacuations from the intestines and urinary bladder, ineffectually, the patient died on the 18th of November, when he had been under medical treatment nineteen days. In this time he took 72 grains of calomel, ʒij of gamboge, ℥iij of oleum ricini, ℥j of jalap, ℥iv of sulphate of magnesia, and ℥iv of quicksilver.

XII. *Observations on the Advantages arising, in some Cases, by bringing on premature Labour.* By WILLIAM SANKEY, Surgeon.

The safety of this practice, when the operation is performed with care and judgment, and its occasional advantages, seems to be fully established in modern practice. Mr. Sankey has added to confidence by the detail of several successful cases in his own practice; but he candidly admits, and we entirely agree with him, that it cannot be decided whether the operation was absolutely necessary in any of these cases. It will be satisfactory to know, however, that the children were all born alive, and the mothers recovered without accident. In about three days after the evacuation of the *liquor amnii*, labour came on; but in one case fifteen days elapsed, and in that time the woman felt no other than mental uneasiness.

XIII. *Pathological and Practical Observations.*

These observations, written with an imposing air of research, search, making only a part of an article, the continuation of which is promised, we, for the present, pass over, in order to resume it when the whole comes before us.

XIV. *Some Observations in reply to Dr. Edward Percival, on the subject of the Poison of Mercury.* By THOMAS BATEMAN, M.D.

In a statement of a case made by Dr. Bateman, in the Report of the practice of a Dispensary, the symptoms were referred to the deleterious action of mercury, the patient having been exposed to the influence of that metal in his avocation. Dr. Edward Percival, of Dublin, in a critical examination of the report of, and observations on, this case, by Dr. Bateman, refers the phenomena of it to the poison of lead; and infers that the diseases of gilders, &c. conjectured to arise from the absorption of mercury, are actually produced by the lead with which mercury is adulterated.

The object of Dr. Bateman's reply is to prove that the morbid appearances in the class of artificers who work much with quicksilver, are actually produced by the absorption of that metal, and not by the lead, with which it is assumed to be combined. In support of this opinion he cites the authority of Fernelius, Etmuller, and particularly Ramazzini, and shows that the mercury employed by the persons thus affected must be pure, or, if any lead remain or is mixed with it, it is not vaporizable; and that the train of symptoms produced by the absorption of lead, differ in character from the *tremores immedicabiles* resulting from the vapor of quicksilver.

We are disposed to think with Dr. Bateman, that a series of specific morbid actions are produced by mercury upon those who work with that metal, especially as gilders; but that they may, and are, often confounded with those arising from the absorption of lead.

XV. *On the Use of Purgatives in Purpura.* By WILLIAM HARTY, M.D.

This is a paper of considerable interest, inasmuch as it states a successful mode of practice, adopted in a disease, where, *à priori*, it would be deemed injurious and hazardous. In the two forms of purpura, the simplex and the hæmorrhagica, as depending, it might be presumed, on debility in the solids, and a dissolved state of the blood, nutritious diet and tonic remedies were indicated. Dr. Harty found, however, this practice unavailing, while the use of purges was successful in the most unpromising cases; and remarkably so in a delicate female, worn down by repeated parturition, poor diet, bad air, deficient clothing, want of cleanliness, and confinement to a cold damp floor,

